# Supplementary material for: Non-invasive quantification of the mitochondrial redox state in livers during machine perfusion
Source: PLoS One. 2021 Oct 27;16(10):e0258833. doi: 10.1371/journal.pone.0258833 (PMC8550443; doi:10.1371/journal.pone.0258833)
Supplement: S1 Fig — a) Vascular resistance in the portal vein. b) Flowrate of the portal vein. c) pressure in the portal vein. d) Weight gain at the end of perfusion compared to the liver weight after procurement. e) Oxygen uptake rate (OUR) f) potassium concentrations in the intrahepatic inferior vena cava. Dots: means. Error bars: SEM. Boxes: Median with interquartile range. Whiskers: min & max. Cones: individual data points. (DOCX) [file pone.0258833.s001.docx]

**f**

**e**

**d**

**c**

**b**

**a**

**S1 Fig. Perfusion parameters of cold ischemic livers.** a) Vascular resistance in the portal vein. b) Flowrate of the portal vein. c) pressure in the portal vein. d) Weight gain at the end of perfusion compared to the liver weight after procurement. e) Oxygen uptake rate (OUR) f) potassium concentrations in the intrahepatic inferior vena cava. Dots: means. Error bars: SEM. Boxes: median with interquartile range. Whiskers: min & max. Cones: individual data points.
