# Supplementary material for: Non-invasive quantification of the mitochondrial redox state in livers during machine perfusion
Source: PLoS One. 2021 Oct 27;16(10):e0258833. doi: 10.1371/journal.pone.0258833 (PMC8550443; doi:10.1371/journal.pone.0258833)
Supplement: S2 Fig — To ascertain the effect on 3RMR measurements via a method of injury other than cold storage, we performed three experiments where rat livers were subjected to 1 hour of warm ischemia in a saline solution followed by 2 hours SNMP. a) A similar trend in 3RMR measurements upon injury followed by recovery as seen for cold ischemic livers. b) The high values for 3RMR at t = 0 min. c) Low values of 3RMR at t = 120 min. d) Oxygen uptake rate (OUR) over the duration of perfusion. e) 3RMR versus OUR. The line shows the trend. f) Potassium concentration. Dots: means. Error bars: SEM. Bar graph: Mean. Cones: individual data points. (DOCX) [file pone.0258833.s002.docx]

**c**

**a**

**b**

p=0.1432

r= -0.3967

**d**

**e**

**f**

**S2 Fig. 3RMR measurements and perfusion parameters of Warm Ischemic livers.** To ascertain the effect on 3RMR measurements via a method of injury other than cold storage, we performed three experiments where rat livers were subjected to 1 hour of warm ischemia in a saline solution followed by 2 hours SNMP. a) A similar trend in 3RMR measurements upon injury followed by recovery as seen for cold ischemic livers. b) The high values for 3RMR at t=0 min. c) Low values of 3RMR at t=120 min. d) Oxygen uptake rate (OUR) over the duration of perfusion. e) 3RMR versus OUR. The line shows the trend. f) Potassium concentration. Dots: means. Error bars: SEM. Bar graph: Mean. Cones: individual data points.
