# Supplementary material for: Non-invasive quantification of the mitochondrial redox state in livers during machine perfusion
Source: PLoS One. 2021 Oct 27;16(10):e0258833. doi: 10.1371/journal.pone.0258833 (PMC8550443; doi:10.1371/journal.pone.0258833)
Supplement: S3 Fig — Primary rat hepatocytes cultured in a sandwich format with collagen I gel show a marginal drop in the mitochondrial membrane potential (MMP) after 24 hours of cold ischemic storage and 3 hours of recovery when compared to fresh hepatocytes, as opposed to a highly significant drop in MMP after 72 hours of cold ischemic storage and 3 hours of recovery. Hepatocytes treated with Carbonyl cyanide 3-chlorophenylhydrazone (CCCP) which disrupts the mitochondrial membrane are used as a positive control. Each bar is for n = 3. (* p = 0.0278; **** p<0.0001). (DOCX) [file pone.0258833.s003.docx]

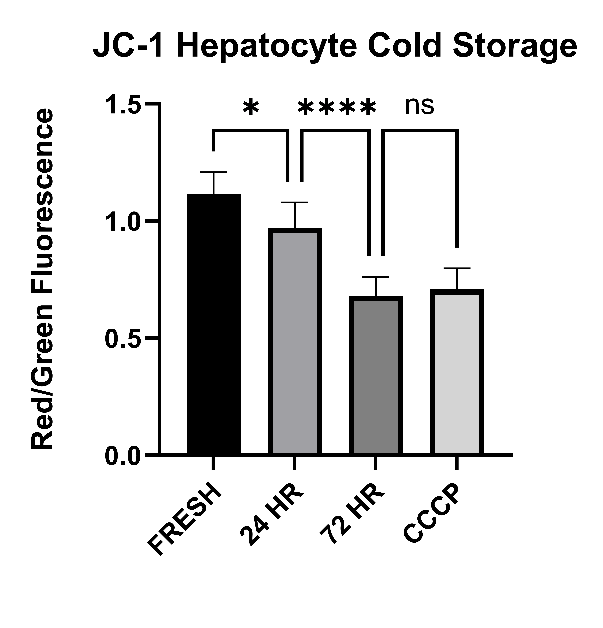


**S3 Fig. Mitochondrial membrane potential in sandwich cultures using JC-1 dye.** Primary rat hepatocytes cultured in a sandwich format with collagen I gel show a marginal drop in the mitochondrial membrane potential (MMP) after 24 hours of cold ischemic storage and 3 hours of recovery when compared to fresh hepatocytes, as opposed to a highly significant drop in MMP after 72 hours of cold ischemic storage and 3 hours of recovery. Hepatocytes treated with Carbonyl cyanide 3-chlorophenylhydrazone (CCCP) which disrupts the mitochondrial membrane are used as a positive control. Each bar is for n=3. (* p=0.0278; **** p<0.0001)
