# Supplementary material for: Non-invasive quantification of the mitochondrial redox state in livers during machine perfusion
Source: PLoS One. 2021 Oct 27;16(10):e0258833. doi: 10.1371/journal.pone.0258833 (PMC8550443; doi:10.1371/journal.pone.0258833)
Supplement: S4 Fig — a) Malondialdehyde (MDA) concentration in tissue biopsies from 24-Hr and 72-Hr cold storage and 3-hour perfusion rat livers. b) Protein carbonyl concentration in tissue biopsies from 24-Hr and 72-Hr cold storage and 3-hour perfusion rat livers. p values of 0.3098 and 0.4751 for MDA level and protein carbonyl content between the two treatment conditions indicate a statistically non-significant difference. Boxes: median with interquartile range. Whiskers: min & max. (DOCX) [file pone.0258833.s004.docx]

**b**

**a**

**S4 Fig. Lipid peroxidation and protein oxidation assays.** a) Malondialdehyde (MDA) concentration in tissue biopsies from 24-Hr and 72-Hr cold storage and 3-hour perfusion rat livers. b) Protein carbonyl concentration in tissue biopsies from 24-Hr and 72-Hr cold storage and 3-hour perfusion rat livers. p values of 0.3098 and 0.4751 for MDA level and protein carbonyl content between the two treatment conditions indicate a statistically non-significant difference. Boxes: median with interquartile range. Whiskers: min & max.
