# Supplementary material for: Non-invasive quantification of the mitochondrial redox state in livers during machine perfusion
Source: PLoS One. 2021 Oct 27;16(10):e0258833. doi: 10.1371/journal.pone.0258833 (PMC8550443; doi:10.1371/journal.pone.0258833)
Supplement: Attachment — Submitted filename: de Vries et al PlosOne_rev_.docx [file pone.0258833.s005.docx]

In the present manuscript de Vries *et al.* investigated the capability of resonance Raman spectroscopy to measure mitochondrial redox status in livers during oxygenated machine perfusion to assess liver damage in a rapid, non-invasive and label-free way. IRI is a major consideration for liver surgery and remains a critical problem for transplantation contributing to limited availability of transplantable livers, therefore finding new diagnostics to identify transplantable organs remain crucial. In this study they adapted and translated the heart specific 3RMR (resonance Raman reduced mitochondrial ratio) and showed that this diagnostic modality can distinguish transplantable versus non-transplantable ischemic rat livers during subnormothermic machine perfusion after cold ischemia.

The authors should be commended for adapting the 3RMR to accurately explain the complex whole liver spectrum with only 4 % remaining residual random error.

As for the formal aspects, the manuscript is logical, well-written and readable, however, there are some typos that should be corrected in the final paper.

The findings are interesting and open novel perspectives to determine donor liver quality. Some details, however, need to be clarified:

1. About what percent of transplanted livers are rejected do to IRI? Percent would be informative in introduction.
2. The numbers in Figure 2a represent characteristic peaks in Raman spectrum where shifts are detected. Numbers 1168 and 1333 are in the wrong order. Can we attribute the shifted peaks to specific molecules? Describe in figure legend if known molecules.
3. Have the authors analyzed mass spectrometry data of flash frozen tissue samples to identify other abundant molecules in rat liver that could exhibit substantial resonance enhancement and would contribute to 3RMR at 441 excitation wavelength?
4. On Suppl. Figure S1. a-f) labeling is missing from the graph. On graph d) weight gain at the end of perfusion show individual dots as on other Figures.
