# Supplementary material for: Non-invasive quantification of the mitochondrial redox state in livers during machine perfusion
Source: PLoS One. 2021 Oct 27;16(10):e0258833. doi: 10.1371/journal.pone.0258833 (PMC8550443; doi:10.1371/journal.pone.0258833)
Supplement: Attachment — Submitted filename: Response to Reviewers.docx [file pone.0258833.s006.docx]

Dear Dr. Tessier,

Thank you for submitting your manuscript to PLOS ONE. After careful consideration, we feel that it has merit but does not fully meet PLOS ONE’s publication criteria as it currently stands. Therefore, we invite you to submit a revised version of the manuscript that addresses the points raised during the review process.

Your manuscript was reviewed by two experts, and we received positive feedback. However, both of them asked few sound questions , which require your attention during revision.

Please submit your revised manuscript by May 10 2021 11:59PM. If you will need more time than this to complete your revisions, please reply to this message or contact the journal office at plosone@plos.org. When you're ready to submit your revision, log on to https://www.editorialmanager.com/pone/ and select the 'Submissions Needing Revision' folder to locate your manuscript file.

Please include the following items when submitting your revised manuscript:

1. A rebuttal letter that responds to each point raised by the academic editor and reviewer(s). You should upload this letter as a separate file labeled 'Response to Reviewers'.
2. A marked‐up copy of your manuscript that highlights changes made to the original version. You should upload this as a separate file labeled 'Revised Manuscript with Track Changes'.
3. An unmarked version of your revised paper without tracked changes. You should upload this as a separate file labeled 'Manuscript'.

If you would like to make changes to your financial disclosure, please include your updated statement in your cover letter. Guidelines for resubmitting your figure files are available below the reviewer comments at the end of this letter.

If applicable, we recommend that you deposit your laboratory protocols in protocols.io to enhance the reproducibility of your results. Protocols.io assigns your protocol its own identifier (DOI) so that it can be cited independently in the future. For instructions see: http://journals.plos.org/plosone/s/submission-guidelines#loc-laboratory-protocols. Additionally, PLOS ONE offers an option for publishing peer-reviewed Lab Protocol articles, which describe protocols hosted on protocols.io. Read more information on sharing protocols at

<https://plos.org/protocols?utm_medium=editorialemail&utm_source=authorletters&utm_campaign=protocols>.

We look forward to receiving your revised manuscript.

Kind regards,

Partha Mukhopadhyay, Ph.D.

Academic Editor

PLOS ONE

Journal Requirements:

When submitting your revision, we need you to address these additional requirements.

1. Please ensure that your manuscript meets PLOS ONE's style requirements, including those for file naming. The PLOS ONE style templates can be found at https://journals.plos.org/plosone/s/file?id=wjVg/PLOSOne_formatting_sample_main_body.pdf and <https://journals.plos.org/plosone/s/file?id=ba62/PLOSOne_formatting_sample_title_authors_affiliations.pdf>
2. Thank you for stating the following in the Competing Interests section:

'We have read the journal's policy and the authors of this manuscript have the following

competing interests: Drs. de Vries, Uygun, Tessier, and Chen, and Mr. Romfh have

provisional patent applications relevant to this study. Dr. Uygun has a financial interest

in Organ Solutions, a company focused on developing organ preservation technology.

P.R. and P.C. are employees and shareholders of Pendar Technologies. All competing

interests are managed by the MGH and Partners HealthCare in accordance with their

conflict of interest policies, except for P.R. and P.C. who are subject to the Research

Integrity Policy of Pendar Technologies. All other authors do not have competing

interest.'

We note that one or more of the authors are employed by a commercial company: Pendar Technologies.

1. Please provide an amended Funding Statement declaring this commercial affiliation, as well as a statement regarding the Role of Funders in your study. If the funding organization did not play a role in the study design, data collection and analysis, decision to publish, or preparation of the manuscript and only provided financial support in the form of authors' salaries and/or research materials, please review your statements relating to the author contributions, and ensure you have specifically and accurately indicated the role(s) that these authors had in your study. You can update author roles in the Author Contributions section of the online submission form.

Please also include the following statement within your amended Funding Statement.

“The funder provided support in the form of salaries for authors [insert relevant initials], but did not have any additional role in the study design, data collection and analysis, decision to publish, or preparation of the manuscript. The specific roles of these authors are articulated in the ‘author contributions’ section.”

This statement has now been added to the Funding Statement.

If your commercial affiliation did play a role in your study, please state and explain this role within your updated Funding Statement.

3. Please also provide an updated Competing Interests Statement declaring this commercial affiliation along with any other relevant declarations relating to employment, consultancy, patents, products in development, or marketed products, etc.

Within your Competing Interests Statement, please confirm that this commercial affiliation does not alter your adherence to all PLOS ONE policies on sharing data and materials by including the following statement: "This does not alter our adherence to PLOS ONE policies on sharing data and materials.” (as detailed online in our guide for authors http://journals.plos.org/plosone/s/competing‐interests) . If this adherence statement is not accurate and there are restrictions on sharing of data and/or materials, please state these. Please note that we cannot proceed with consideration of your article until this information has been declared.

Please include both an updated Funding Statement and Competing Interests Statement in your cover letter. We will change the online submission form on your behalf.

Both the sections have been added to the cover letter.

4. We note that you have a patent relating to material pertinent to this article. Please provide an amended statement of Competing Interests to declare this patent (with details including name and number), along with any other relevant declarations relating to employment, consultancy, patents, products in development or modified products etc. Please confirm that this does not alter your adherence to all PLOS ONE policies on sharing data and materials, as detailed online in our guide for authors http://journals.plos.org/plosone/s/competing‐interests by including the following statement:

"This does not alter our adherence to PLOS ONE policies on sharing data and materials.” If there are restrictions on sharing of data and/or materials, please state these. Please note that we cannot proceed with consideration of your article until this information has been declared.

This information has now been included.

This information should be included in your cover letter; we will change the online submission form on your behalf.

Please know it is PLOS ONE policy for corresponding authors to declare, on behalf of all authors, all potential competing interests for the purposes of transparency. PLOS defines a competing interest as anything that interferes with, or could reasonably be perceived as interfering with, the full and objective presentation, peer review, editorial decision‐making, or publication of research or non‐research articles submitted to one of the journals. Competing interests can be financial or non‐financial, professional, or personal. Competing interests can arise in relationship to an organization or another person. Please follow this link to our website for more details on competing interests:

<http://journals.plos.org/plosone/s/competing‐interests>

4. Please include captions for your Supporting Information files at the end of your manuscript, and update any in‐text citations to match accordingly. Please see our Supporting Information guidelines for more information:

http://journals.plos.org/plosone/s/supporting‐information.

Additional Editor Comments (if provided):

[Note: HTML markup is below. Please do not edit.]

Reviewers' comments:

Reviewer's Responses to Questions

**Comments to the Author**

1. Is the manuscript technically sound, and do the data support the conclusions?

The manuscript must describe a technically sound piece of scientific research with data that supports the conclusions. Experiments must have been conducted rigorously, with appropriate controls, replication, and sample sizes. The conclusions must be drawn appropriately based on the data presented.

Reviewer #1: Partly

Reviewer #2: Yes

2. Has the statistical analysis been performed appropriately and rigorously?

Reviewer #1: Yes

Reviewer #2: Yes

3. Have the authors made all data underlying the findings in their manuscript fully available?

The PLOS Data policy requires authors to make all data underlying the findings described in their manuscript fully available without restriction, with rare exception (please refer to the Data Availability Statement in the manuscript PDF file). The data should be provided as part of the manuscript or its supporting information, or deposited to a public repository. For example, in addition to summary statistics, the data points behind means, medians and variance measures should be available. If there are restrictions on publicly sharing data—e.g. participant privacy or use of data from a third party—those must be specified.

Reviewer #1: Yes

Reviewer #2: Yes

4. Is the manuscript presented in an intelligible fashion and written in standard English?

PLOS ONE does not copyedit accepted manuscripts, so the language in submitted articles must be clear, correct, and unambiguous. Any typographical or grammatical errors should be corrected at revision, so please note any specific errors here.

Reviewer #1: Yes

Reviewer #2: Yes

5. Review Comments to the Author

Please use the space provided to explain your answers to the questions above. You may also include additional comments for the author, including concerns about dual publication, research ethics, or publication ethics. (Please upload your review as an attachment if it exceeds 20,000 characters)

Reviewer #1: The present study “Non‐invasive Quantification of the Mitochondrial Redox State in Livers during Machine Perfusion” is a pioneer discussion about a new method for evaluating liver graft viability. It has good practical merit.

We thank the reviewer for careful consideration of the manuscript and thoughtful comments that have improved the manuscript. We provide a point-by-point response to all comments below:

Here are some major concerns to address:

1. The study compared short and long preserved livers as viable and unviable grafts. However, preservation itself may change the mitochondrial integrity. The results in this study are thus only indicative of the length of preservation. To better define the methods provided by this study, the authors need to compare a well-preserved graft with an inviable liver graft destroyed in at least another way.

**Response**: To assess 3RMR with another form of injury that is different from cold ischemia, we performed an experiment where rat livers were incubated at 37° C for 1 hour in saline solution. This treatment is expected to cause substantial warm ischemic injury to the livers as has been shown by our group as well as others before^1,2^. This treatment is followed by machine perfusion and 3RMR measurements of the warm ischemic (WI) livers. The 3RMR values and perfusion parameters are as shown below.

p=0.1432

r= -0.3967

**c**

**b**

**d**

**f**

**e**

**a**

We show high 3RMR values immediately after inducing warm ischemic injury to the liver, indicating the change in 3RMR is not specific to preservation modality. As expected, the perfusion parameters recover over time suggesting that perfusion of these livers has a beneficial effect.

We thank the reviewer for this important experiment and have now included it in Supplementary Figure 2 and lines 168-172 as an additional method of injury to the liver and recovery as observed by 3RMR measurements.

2. The authors removed the interference from carotene and bile. How do the authors decide the amplitude of these parameters (the beta constants in Figure 1)?

However, lab rats have very consistent living condition to make these two parameters almost the same between different individuals. Plus, there is no gallbladder, which contributes to the consistency of bile content in the liver. Human do not live in such conditions and everyone’s diet will change these settings. How do the authors provide an accurate array of parameters to apply to each liver graft?

**Response**: The reviewer is referring to the algorithm that is used to identify relative contributions of different molecular entities in the overall Raman spectra from the rat liver tissue. Molecular components of the liver that are expected to produce significant Raman scattering with 441 nm excitation laser are identified from the literature and their unique Raman scattering signature is characterized in a calibration step (please see lines 121-143, and 399-417). Then the fitting algorithm tries to fit these calibrated spectra on a Raman spectrum from a liver sample with unknown concentrations of each of these molecules. Just as the relative concentration of oxidized and reduced mitochondria will vary between individuals as well as time to time, the relative concentration of molecules such as carotene and bile will also vary significantly depending on the diet, environment, or genetics of every individual. Because the algorithm is used for fitting the spectrum every time an experiment is performed, the differences in concentrations would lead to different values of all beta constants included in the library as defined in Figure 1. Thus, the relative concentrations of oxidized mitochondria as well reduced mitochondria are accurately measured even when there are differences in concentrations of the other molecules between subjects.

We thank the reviewer for this important clarification. We have now expanded this procedure in the results section to describe it better (lines 121-129).

3. The authors explained the present method as a measurement of mitochondrial redox condition. However, Figure 4B did not show a significance between viable and unviable grafts in mitochondrial function. This is a major inconsistency. The authors need to explain it.

Also, the authors need directly measure the mitochondrial function and membrane potential whether or not it is invasive.

**Response**: Figure 4B of the manuscript shows a comparison of oxygen uptake during perfusions of the rat livers for 24- and 72-hours of cold storage. The reviewer makes an excellent point – oxygen is not a direct measurement of mitochondrial function and membrane potential and can be influenced by many complex factors. Hence, to augment this analysis and provide a more direct indicator of mitochondrial function, we added data that measure the mitochondrial membrane potential (MMP) using the JC-1 assay^3^ on plated primary rat hepatocytes exposed to 24- and 72-hours of cold storage. This result is now also included in Supplementary Figure 3 of our manuscript (also shown below). It is observed that the red/green fluorescence ratio which is directly proportional to MMP and indicative of healthy metabolic activity, is highest in fresh controls and marginally goes down (p=0.0278) in 24-hour cold ischemic hepatocytes. It is significantly lower (p<0.0001) for the 72-hour cold ischemic hepatocytes that is comparable to the positive/CCCP controls, indicating the poor metabolic activity of these hepatocytes. This result closely corresponds to the significant difference in 3RMR values at the end of 3 hours of perfusion in rat liver after 24- and 72-hours of cold storage.

We thank the reviewer for this important consideration which was not addressed before and has now been discussed in lines 218-224 and 306-311 of our revised manuscript.

*-p=0.0278, ****- p<0.0001

4. The authors should analyze lipid or protein peroxidation levels in Figure 5.

**Response**: We used tissue biopsies to measure Malondialdehyde (MDA) and Protein Carbonyl levels which are established markers of lipid peroxidation^6^ and protein oxidation^7^ levels respectively. The Lipid Peroxidation (MDA) Assay Kit (MAK085) by Millipore Sigma and the Protein Carbonyl Content Assay Kit (ab126287) by Abcam were used according to the manufacturer’s instructions. The livers that were stored for 24 hours versus 72 hours did not show any statistically significant difference in either their MDA levels (p=0.3098) or their protein carbonyl content (p=0.4751). This result concurs with the other measures of cellular oxidation state- NAD: NADH and NADP: NADPH values as shown in the manuscript. We hypothesize this is because 3RMR is a more direct measure of mitochondrial redox state, while lipid and protein peroxidation levels can be influenced by several factors. We have now also included these results as Supplementary Figure 4 and discussed them in lines 236-241.

Reviewer #2: In the present manuscript de Vries et al. investigated the capability of resonance Raman spectroscopy to measure mitochondrial redox status in livers during oxygenated machine perfusion to assess liver damage in a rapid, non‐invasive and label‐free way. IRI is a major consideration for liver surgery and remains a critical problem for transplantation contributing to limited availability of transplantable livers, therefore finding new diagnostics to identify transplantable organs remain crucial.

In this study they adapted and translated the heart specific 3RMR (resonance Raman reduced mitochondrial ratio) and showed that this diagnostic modality can distinguish transplantable versus non-transplantable ischemic rat livers during sub-normothermic machine perfusion after cold ischemia.

The authors should be commended for adapting the 3RMR to accurately explain the complex whole liver spectrum with only 4 % remaining residual random error.

As for the formal aspects, the manuscript is logical, well‐written and readable, however, there are some typos that should be corrected in the final paper.

The findings are interesting and open novel perspectives to determine donor liver quality. Some details, however, need to be clarified.

We thank the reviewer for careful consideration of the manuscript and thoughtful comments that have improved the manuscript. We provide a point-by-point response to all comments below:

1. About what percent of transplanted livers are rejected do to IRI? Percent would be informative in introduction.

**Response**: We agree with the reviewer as adding these numbers would provide more context to the problem our device is trying to solve. The following information from the literature better defines the scope of the problem and has now also been added to the manuscript in lines 42-48.

While the total number of liver donations after brain death (DBD) and donations after circulatory death (DCD) have increased over the years, about 7.1% of DBD livers and 30% of DCD livers are discarded. As many as 58.5% of DCD discards meet the standard acceptance criteria proposed by the American Society of Transplant Surgeons^8^. Many waitlisted patients die or become too sick to undergo transplantation, representing several thousand deaths a year in the US alone^9,10^. For every 4 patients that are removed from the transplant waiting list due to death or illness, 3 unused livers are discarded^11^.

2. The numbers in Figure 2a represent characteristic peaks in Raman spectrum where shifts are detected. Numbers 1168 and 1333 are in the wrong order. Can we attribute the shifted peaks to specific molecules? Describe in figure legend if known molecules.

**Response**: We thank the reviewer for pointing out this error and have now replaced the old Figure 2a with a new Figure 2a that corrects this error as shown below.

The marked peaks represent the most significant contributions from the different molecules. However, a given molecule may have contributions to more than one part of the spectrum. Hence, the regression algorithm uses the library spectrum of each molecule to best explain the measured unknown spectrum across the entire range rather than identifying individual peaks. To showcase the contribution from an individual molecule, we have included the library spectrum of each component (x_1_ to x_5_) in Figure 1. These individual components are assigned weights based on the regression algorithm and get added according to those weights to explain the overall Raman spectrum from a liver as discussed in the newly added lines 121-129 of the manuscript. According to the reviewer’s recommendation, we have also now identified each of the components x_1_ to x_5_ with their associated molecule in the caption of Figure 1. We thank the reviewer for their recommendation on this important detail.

3. Have the authors analyzed mass spectrometry data of flash frozen tissue samples to identify other abundant molecules in rat liver that could exhibit substantial resonance enhancement and would contribute to 3RMR at 441 excitation wavelength?

**Response**: The reviewer is referring to our methodology for identifying molecules other than the reduced and oxidized mitochondria that contribute significantly to the Raman spectrum for a 441 nm excitation wavelength. Our strategy to identify molecules exhibiting substantial resonance enhancement relied on a thorough literature search and a mathematical constraint to minimize the root mean square error of the regression fit (see lines 136-142 of the manuscript). Since our root mean square error was only 4% of the mitochondrial signal, we believe that the current library is sufficient to perform this kind of analysis. However, in the future when we translate this approach to human experimentation, further modifications to this approach as proposed by the reviewer may be necessary. We have now indicated this in our discussion in lines 341-343.

4. On Suppl. Figure S1. a‐f) labeling is missing from the graph. On graph d) weight gain at the end of perfusion show individual dots as on other Figures.

**Response**: We thank the reviewer for pointing out these details that have now been modified in the attached file for supplementary information.

6. PLOS authors have the option to publish the peer review history of their article (what does this mean?). If published, this will include your full peer review and any attached files.

If you choose “no”, your identity will remain anonymous but your review may still be made public.

**Do you want your identity to be public for this peer review?** For information about this choice, including consent withdrawal, please see our Privacy Policy.

Reviewer #1: No

Reviewer #2: No

[NOTE: If reviewer comments were submitted as an attachment file, they will be attached to this email and accessible via the submission site. Please log into your account, locate the manuscript record, and check for the action link "View Attachments". If this link does not appear, there are no attachment files.]

While revising your submission, please upload your figure files to the Preflight Analysis and Conversion Engine (PACE) digital diagnostic tool, https://pacev2.apexcovantage.com/. PACE helps ensure that figures meet PLOS requirements. To use PACE, you must first register as a user. Registration is free. Then, login and navigate to the UPLOAD tab, where you will find detailed instructions on how to use the tool. If you encounter any issues or have any questions when using PACE, please email PLOS at figures@plos.org. Please note that Supporting Information files do not need this step.

*In compliance with data protection regulations, you may request that we remove your personal registration details at any time. (Remove my information/details). Please contact the publication office if you have any questions.*

1.	Hide, D. *et al.* Effects of warm ischemia and reperfusion on the liver microcirculatory phenotype of rats: underlying mechanisms and pharmacological therapy. *Sci. Reports 2016 61* **6**, 1–12 (2016).

2.	Bruinsma, B. G. *et al.* Warm Ischemic Injury Is Reflected in the Release of Injury Markers during Cold Preservation of the Human Liver. *PLoS One* **10**, e0123421 (2015).

3.	Sivandzade, F., Bhalerao, A. & Cucullo, L. Analysis of the Mitochondrial Membrane Potential Using the Cationic JC-1 Dyeas a Sensitive Fluorescent Probe. *Bio-protocol* **9**, (2019).

4.	Dunn, J. C. Y., Tompkins, R. G. & Yarmush, M. L. Long‐Term in Vitro Function of Adult Hepatocytes in a Collagen Sandwich Configuration. *Biotechnol. Prog.* **7**, 237–245 (1991).

5.	Dunn, J. C. Y., Yarmush, M. L., Koebe, H. G. & Tompkins, R. G. Hepatocyte function and extracellular matrix geometry: long-term culture in a sandwich configuration.

6.	S, G., M, W., E, N. & P, W. [Malondialdehyde (MDA) as a lipid peroxidation marker]. *Wiad. Lek.* **57**, 453–455 (2004).

7.	Dalle-Donne, I., Rossi, R., Giustarini, D., Milzani, A. & Colombo, R. Protein carbonyl groups as biomarkers of oxidative stress. *Clin. Chim. Acta* **329**, 23–38 (2003).

8.	Haque, O., Yuan, Q. & Markmann, J. Evolving utilization of donation after circulatory death livers in liver transplantation: The day of DCD has come. *Clin. Transplant.* **35**, 5 (2021).

9.	WR, K. *et al.* OPTN/SRTR 2017 Annual Data Report: Liver. *Am. J. Transplant* **19 Suppl 2**, 184–283 (2019).

10.	de Vries, R. J. *et al.* Cell release during perfusion reflects cold ischemic injury in rat livers. *Sci. Rep.* **10**, (2020).

11.	S, G. *et al.* The promise of organ and tissue preservation to transform medicine. *Nat. Biotechnol.* **35**, 530–542 (2017).
